# Measurement of pesticides in hair samples from pemphigus foliaceus and pemphigus vulgaris patients in Southeastern Brazil^☆^

**DOI:** 10.1016/j.abd.2022.10.010

**Published:** 2023-05-17

**Authors:** Leonardo La Serra, Adriana Martinelli Salathiel, Rafael Lanaro, Bruno de Martinis, Ana Maria Roselino

**Affiliations:** a Department of Internal Medicine, Division of Dermatology, University Hospital, Faculty of Medicine of Ribeirão Preto, Ribeirão Preto, SP, Brazil; b Poison Control Center, Faculty of Medical Sciences, State University of Campinas, Campinas, SP, Brazil; c Faculty of Pharmaceutical Sciences, State University of Campinas, Campinas, SP, Brazil; d Department of Chemistry, Faculty of Philosophy, Sciences and Letters of Ribeirão Preto, University of São Paulo, Ribeirão Preto, SP, Brazil

**Keywords:** Diazinon, dichlorvos, Hydrocarbons, chlorinated, Organophosphate, Pemphigus, Pesticides

## Abstract

**Background:**

Pesticides, mainly organophosphates (OP), have been related to increased risk of pemphigus vulgaris (PV) and pemphigus foliaceus (PF), nevertheless, their measurement has not been determined in pemphigus patients.

**Objective:**

To evaluate pesticide exposure and pesticide measurement, comparing PV, PF and control groups in Southeastern Brazil.

**Methods:**

Information about urban or rural residency and exposure to pesticides at the onset of pemphigus was assessed by questionnaire interview; hair samples from the scalp of PV, PF, and controls were tested for OP and organochlorines (OC) by gas-phase chromatography coupled to mass spectrometry.

**Results:**

The minority of PV (2 [7.1%] of 28) and PF (7 [18%] of 39), but none of the 48 controls, informed living in rural areas at the onset of pemphigus (p = 0.2853). PV (33.3%), PF (38.5%), and controls (20%) informed exposure to pesticides (p = 0.186). Twenty-one (14.8%) of 142 individuals tested positive for OP and/or OC: PV (2 [6.3%] of 32) and PF (11 [25.6%] of 43) had similar pesticides contamination as controls (8 [11.9%] of 67) (p = 0.4928; p = 0.0753, respectively), but PF presented higher contamination than PV (p = 0.034). PV did not present any positivity for OP. Three (7%) PF tested positive for both OP and OC. Some PF tested positive for three or four OP, mainly diazinon and dichlorvos.

**Study limitation:**

Lack of data for some controls.

**Conclusion:**

Although the frequency of PV and PF patients exposed to pesticides was similar, pesticides were more frequently detected in hair samples from PF compared to PV. The cause-effect relationship still needs to be determined.

## Introduction

Pemphigus encompasses a group of autoimmune bullous diseases resulting from the production of autoantibodies against Desmogleins (Dsg). IgG autoantibodies against Dsg1 affect the skin in pemphigus foliaceus (PF), and IgG autoantibodies against Dsg3 and Dsg1 affect mucous membranes and the skin in pemphigus vulgaris (PV).^1^ PF is endemic in Brazil, where it is known as “fogo selvagem”. More recently, PV diagnosis surpassed PF diagnosis in some Brazilian regions.^2^, ^3^ While HLA class I and II alleles differentiate PF from PV clinical forms, their etiopathogenesis is not fully understood.^4^, ^5^, ^6^

Exogenous factors have been associated with pemphigus pathogenesis, including drugs and nutritional elements rich in thiol, heavy metals, herpes virus infection, and insect bite.^7^, ^8^, ^9^, ^10^, ^11^, ^12^, ^13^, ^14^ Furthermore, in case-control studies based on interviews, exposure to pesticides, mainly organophosphates (OP), has been related to PV in Asia, Europe, and North America,^15^, ^16^, ^17^, ^18^, ^19^ and also to “fogo selvagem” in Brazil.^20^ PV following contact dermatitis with dihydrodiphenyltrichlororethane and diazinon has been described.^21^, ^22^ By using normal human skin as substrate and PV serum samples, chlorpyrifos induced pemphigus in an in vitro experiment.^23^

There is no report of a larger approach regarding pesticide measurement in a wider Brazilian pemphigus population for a better understanding of its environmental link to pemphigus. This study aimed to evaluate exposure to pesticides by means of (i) A questionnaire-based interview and (ii) Quantitative measurement of OP and organochlorines (OC) in scalp hair samples from PV and PF patients, and controls living in the northeastern region of the state of São Paulo, a southeastern Brazilian region where both clinical forms of pemphigus are prevalent.^3^

## Materials and methods

In this case-control transversal study, three groups were compared: PV patients (designated PV hereafter), PF patients (designated PF hereafter), and controls. This study was approved by the local Committee for Ethics in Research of the University Hospital of Ribeirão Preto Medical School of the University of São Paulo (# 2248/2010), and all the participants provided an informed written consent form.

### Groups of patients and controls

Seventy-five pemphigus patients assisted at the Dermatological Outpatient Clinic of the University Hospital of Ribeirão Preto Medical School, University of São Paulo, Brazil, were enrolled in the study. Among them, 32 and 43 had PV (median age = 50 years, 75% female) and PF (median age = 46 years, 58.1% female), respectively (Table 1). PV and PF diagnosis was based on clinical, histopathological, and immunofluorescence (direct, indirect, or both) features and determination of autoantibodies against Dsg1 and Dsg3 by ELISA (MBL, Japan). Controls consisted of 67 healthy pemphigus patients’ relatives and neighbors (median age = 46 years, 70.1% female) (Table 1). PV, PF, and controls lived in the northeastern region of the state of São Paulo, southeastern Brazil.

**Table 1:** Demographic and clinical data, area of residency and exposure to pesticides on the onset of disease, recorded in questionnaires, and organophosphate and organochlorine detection in hair samples from pemphigus vulgaris (PV) and pemphigus foliaceus (PF) patients and Controls

		PV	PF	Controls	p-value
		n = 32	n = 43	n = 53	
**Age (years) Median (p25–p75)**		50 (36–62)	46 (31–58)	46 (38–59)	0.6513
		**n =** **32 (%)**	**n =** **43 (%)**	**n =** **67 (%)**	
**Gender**	Male	8 (25.0)	18 (41.9)	20 (29.9)	0.2218
	Female	24 (75.0)	25 (58.1)	47 (70.1)	
		**n =** **31**	**n =** **43**	NA	
**Disease duration (years) Median (p25–p75)**		4 (2–6)	7 (5–14)		*0.005*
Questionnaire interview*					
		**n =** **28 (%)**	**n =** **39 (%)**	**n =** **48 (%)**	^a,b^0.2853
**Area of residency**	Rural	2 (7.1)^a^	7 (18.0)^b^	0 (0)	
	Urban	26 (92.9)	32 (82.0)	48 (100)	
		**n =** **27 (%)**	**n =** **39 (%)**	**n =** **40 (%)**	0.1860
**Exposure to Pesticides**	Yes	9 (33.3)	15 (38.5)	8 (20.0)	
	No	18 (66.7)	24 (61.5)	32 (80.0)	
**Pesticides hair detection**		**n =** **32 (%)**	**n =** **43 (%)**	**n =** **67 (%)**	
Organophosphates	Yes	0 (0)	9 (20.9)^b^	6 (9.0)^c^	^b,c^0.0914
	No	32 (100)	34 (79.1)	61 (91.0)	
Organochlorines	Yes	2 (6.3)	5 (11.6)	2 (3.0)	0.1707
	No	30 (93.7)	38 (88.4)	65 (97.0)	
Total	Yes	2 (6.3)^a^	11 (25.6§^b^	8 (11.9)^c^	^abc^*0.043*
					^ab^*0.034*
	No	30 (93.7)	32 (74.4)	59 (88.1)	^bc^0.0753
					^ac^0.4928

NA, Not Applicable.

* Individuals in the PV and control groups answered the questionnaire disproportionately.

§ Three PF patients had both organophosphate and organochlorine detection.

### Questionnaires

The questionnaires, designed by the authors, were applied to 28 PV and 39 PF patients, and to 48 controls. The answers to each item of the questionnaires were incomplete amongst the three groups. Thus, 28 PV patients and 40 controls answered the “Area of residency”; and 27 PV patients and 48 controls answered the “Exposure to pesticides” questions (Table 1). The subjects were interviewed per occasion of their visit to the Dermatological Outpatient Clinic of the University Hospital of Ribeirão Preto Medical School, University of São Paulo, Brazil. Two main pieces of information were analyzed: area of residency (rural or urban) per occasion of the onset of pemphigus and exposure to pesticides related to professional activities or other causes.

### Hair sample collection

With the aid of a sterile scissor, nearly 500 mg of hair was cut from the root scalp in the occipital area and stored in individual paper envelopes at room temperature until analysis. The samples were tested for OP and OC pesticides.

### Organophosphate and organochlorine analytical methods and acceptable values

The pesticides belonging to the OP and OC classes were analyzed by Gas-phase Chromatography coupled with Mass Spectrometry (GC/MS). The extraction procedure for OP and OC hair analysis was the same as the one described by Tsatsakis et al.^24^ and adapted to the instrumental conditions of the Laboratory of Forensic Toxicology of the Department of Chemistry of the Faculty of Philosophy, Sciences and Letters at Ribeirão Preto, University of São Paulo, Brazil. The experiments were carried out on a 7890A gas chromatograph coupled to a 5975C Inert mass spectrometer (Agilent Technologies, USA), equipped with a DB-5MSUI (30 m × 0.25 mm/i.d., 0.25 µm film thickness) chromatographic column (Agilent Technologies, USA). The analytes were eluted with oven linear programming temperature, as described below: 60 °C for 2.0 min, increase to 315 °C at 30 °C.min^-1^, and 315 °C for 2 min (total time of 12.5 min). The injection port and transfer line temperatures were set to 280 °C. Ultrapure helium was used as carrier gas at a flow rate of 1.0 mL.min^-1^, and the samples were injected in the splitless mode (ultra-inert deactivated split liner with glass wool was used to improve chromatography). The mass spectrometer was set to SIM mode. The data were acquired, and the instrument was controlled with the Agilent Technologies ChemStation software, version E02021431, supplied by the manufacturer. Supplementary Table 1 provides the LOD/LOQ of the pesticides. The Acceptable Daily Intake (ADI) values for the OP detected in this study are as follows: chlorpyrifos (10 pg/mg), diazinon (2 pg/mg), and malathion (300 pg/mg).^25^ The use of dichlorvos, methyl parathion, and parathion has been banned. The use of OC aldrin, BHC, DDT, and endrin has also been banned, so no ADI values are available for them. The samples that tested positive but below the limit of detection (< LOQ = Limit of Quantification) were included in our study.

### Statistical analysis

Kruskall-Wallis and Mann-Whitney tests were used to analyze the age and duration of the disease, respectively. Fischer’s test was used to analyze variable frequencies; p ≤ 0.05 was adopted, and a two-tailed comparison was employed. Statistical analysis was performed by using the SPSS 26.0 software (IBM, USA).

## Results

### Demographic and clinical data

The studied groups composed of 32 PV, 43 PF, and controls did not differ in terms of age or gender. Age data from 14 of 67 controls were not assessed. Disease lasted longer in PF than in PV (p = 0.005) (Table 1).

### Questionnaire-based interview data

There was no statistical difference comparing the urban or rural area of residency among PV, PF, and controls (p = 0.2853). Two (7.1%) of 28 PV and 7 (18%) of 39 PF, but none of the 48 controls, informed living in rural areas. Positive or negative information regarding exposure to pesticides was similar among the groups (p = 0.186) (Table 1).

### OP and OC measurement in hair samples

OP and OC were measured in hair samples from 32 PV, 43 PF, and 67 controls (Table 1). Twenty-one (14.8%) of 142 individuals tested positive for OP, OC, or both. PF (11 [25.6%] of 43) were more contaminated than PV (2 [6.3%] of 32), but as contaminated as controls (8 [11.9%] of 67) (p = 0.034 and p = 0.0753, respectively). PV and controls had similar pesticide detection (p = 0.4928). Three (7%) PF tested positive for OP and OC. None of the 32 PV tested positive for OP.

Four OC and six OP were detected in patients and controls (Table 2). Among OC, aldrin was detected in two controls (< LOQ and 7.7 pg/mg); BHC in two PV and four PF (range ≤ LOQ to 12.2 pg/mg); DDT in one PV and two PF (< LOQ); and endrin in one control (9.1 pg/mg). Among OP, chlorpyrifos was detected in two PF and three controls (range ≤ LOQ to 12.6 pg/mg); diazinon in five PF and two controls (range ≤ LOQ to 15.6 pg/mg); dichlorvos in four PF and two controls (range ≤ LOQ to 9.1 pg/mg); malathion in two PF and one control (range ≤ LOQ to 8.3 pg/mg); methyl parathion in one PF (12.1 pg/mg); and parathion in another PF (< LOQ). Some PF tested positive for three or four OP; diazinon and dichlorvos predominated. Diuron was detected in one PF. Seven (70%) of 10 PF were female, lived in rural areas on the onset of pemphigus, and had been exposed to pesticides during their peasant professional activities (Table 2).

**Table 2:** Demographic and questionnaires data, and classes of pesticides detected in hair samples from pemphigus vulgaris (PV) (n = 32), Pemphigus Foliaceus (PF) (n = 43), and Control (C) (n = 67) groups

	Gender	Disease onset to hair sample collection (years)	Age	Profession	Questionnaires		Classes of pesticides (pg/mL)^a^										
					Residence	Pesticide	Organochlorines				Organophosphates						Other
						Exposure	Aldrin	BHC	DDT	Endrin	Chlorpyriphos	Diazinon	Dichlorvos	Malathion	Methyl parathion	Parathion	
PV_1_	F	4	53	Housemaid	Urban	NA		9.2									
PV_2_	M	5	72	Beekeeper	Rural	Yes		<LOQ	<LOQ								
PF_1_	M	13	58	Peasant	Urban	Yes					<LOQ			8.3			
PF_2_	F	8	32	Peasant	Rural	Yes					12.6	<LOQ	6.4				
PF_3_	F	4	72	Housemaid	Rural	No							5.8				
PF_4_	M	19	49	Peasant	Urban	Yes		12.2				2.1					
PF_5_	M	32	73	Peasant	Rural	Yes			<LOQ				<LOQ				Diuron
PF_6_	F	18	52	Peasant	Rural	Yes		<LOQ	<LOQ			7.6					
PF_7_	F	6	51	Housemaid	Rural	Yes		8.3									
PF_8_	F	9	39	Peasant	Urban	Yes						15.6					
PF_9_	F	0	39	Peasant	Urban	Yes						3.5	9.1		12.1	<LOQ	
PF_10_	F	0	59	Housemaid	Rural	Yes								<LOQ			
PF_11_	F	49	58	NA	Rural	No		7.4									
C_1_	M	NA	58	Tapestry	Urban	No						8.4		5.5			
C_2_	F	NA	NA	NA	NA	NA	7.7			9.1							
C_3_	F	NA	NA	Housemaid	NA	NA	<LOQ										
C_4_	M	NA	47	NA	NA	NA					8.2						
C_5_	F	NA	NA	NA	NA	NA					<LOQ		7.2				
C_6_	M	NA	NA	NA	NA	NA						6.3					
C_7_	F	NA	54	Dressmaker	Urban	Yes					<LOQ						
C_8_	F	NA	53	Housemaid	Urban	No							<LOQ				

NA, Not Available; LOQ, Limit of Quantification; BHC, Benzene Hexachloride; DDT, Dichlorodiphenyltrichloroethane.

a Dimethoate, Terbufos, Disulfoton, Heptachlor, Fenitrothion, Fenthion, Methidathion, Endosulfan, Ethion, Hexazinone, and Ketoendrin were not detected.

## Discussion

Pemphigus pathogenesis is attributed to genetic background and environmental triggers, including pesticides.^4^, ^5^, ^6^, ^15^, ^16^, ^17^, ^18^, ^19^, ^20^, ^26^ There have been few reports of pesticides triggering autoimmunity. They have been described to cause immunotoxicity, endocrine dysfunction, neurodegenerative disorders, and cancer, among other conditions. Regarding autoimmune diseases, pesticides have mainly been related to systemic lupus erythematous and rheumatoid arthritis.^27^, ^28^, ^29^, ^30^

To our knowledge, this is the first report on the measurement of pesticides in PV and PF patients, compared to controls. We have opted by measuring pesticides in hair samples because they are considered human biomarkers of pesticide exposure.^31^, ^32^, ^33^ The present study confirmed contamination with OP and/or OC in hair samples in PV, PF and controls (6.3%, 25.6%, and 11.9%, respectively) (p = 0.0437). Compared to PV, PF presented a higher frequency of contamination with pesticides (p = 0.034). Moreover, 9 (20.9%) of 43 PF, but none of 32 PV tested positive for OP. Some PF tested positive for three or four OP: diazinon and dichlorvos predominated. OC was detected similarly in PV, PF, and controls although its use has been banned since 1985.^34^

The immune mechanism proposed for PV using chlorpyrifos,^23^ which is one of the OP detected in PF and controls in this study, fails to explain PV pathogenesis in our study given that PV did not present any OP contamination.

How can the increased prevalence of PF pesticide contamination be explained? Historically, in Brazil, PF was distributed in rural areas close to rivers, and exposure to pesticides was considered a possible risk factor for PF in this country.^20^, ^35^ Nonetheless, the questionnaire-based interviews revealed no statistical difference among PV, PF, and controls in terms of urban or rural residency and exposure to pesticides. On the other hand, most PF with OC or OP hair contamination informed that they lived in rural areas at the onset of pemphigus and had been exposed to pesticides during their peasant professional activities (Table 2). Furthermore, the disease lasted longer in PF than in PV, which may have contributed to the bioaccumulation of pesticides in PF.

Exposure to pesticides has been confirmed in blood donors and in the rural population in Brazil.^36^, ^37^, ^38^ Moreover, the immunologic profile has been determined in Brazilian farmers exposed to pesticides.^39^, ^40^ Here, of 8 controls who presented contamination with pesticides (6 OP and 2 OC) (Table 2), autoantibodies against Dsg1 and Dsg3 were investigated using ELISA (MBL, Japan) in three (C_1_, C_7_ and C_8_), with negative results. HLA class II profiles were determined in the eight controls (C_1_ to C_8_) (data not shown). Two of 6 controls contaminated with OP presented: one Control (C1) contaminated with diazinon and malathion presented both *HLA*-DRB1*01:01 and *HLA*-DQB1*05:01 alleles of susceptibility to PF, and another one (C5) contaminated with dichlorvos and chlorpyriphos presented *HLA*-DQB1*03:02 allele of susceptibility to PV. The other six controls did not present any associated alleles with PV and PF (for PV and PF-associated *HLA* alleles, see Brochado et al.^4^). Knowing that both clinical forms of pemphigus are prevalent in the northeastern region of the state of São Paulo, Brazil,^35^ testing antibodies against desmogleins and determining *HLA* alleles in a wide population exposed to pesticides would provide important information on the pesticides-pemphigus relationship.

Our results corroborate with a recent report by Chang and Tsai (2022), adopting a systematic review and meta-analysis. They included five case-control studies^15^, ^16^, ^17^, ^19^, ^20^ based on interviews regarding the relationship between pesticide exposure and PV and PF. They concluded to have an association of pesticide exposure with pemphigus.^41^ Here, we demonstrated OC and/or OP hair contamination in the three studied groups, being PF group that presented the highest pesticide contamination.

## Conclusion

The present study confirmed hair contamination with OC and/or OP pesticides in PF, PV, and controls. PV patients did not test positive for OP. Some PF patients tested positive for three or four OP: diazinon and dichlorvos predominated. Although PV and PF provided similar information about living in rural areas on the onset of pemphigus and being exposed to pesticides, the detection of pesticides was more frequent in PF hair samples compared to PV. The pesticide-PF pathogenesis relationship still has to be elucidated.

## Financial support

This study was partially funded by the FAPESP (Fundação de Amparo à Pesquisa do Estado de São Paulo) (#2010/51729-2); the first author received a Master's scholarship from CAPES (Coordenação de Aperfeiçoamento de Pessoal de Nível Superior).

## Authors' contributions

Bruno de Martins: Approval of the final version of the manuscript; collection, analysis, and interpretation of data; critical review of the manuscript.

Leonardo La Serra: Statistical analysis; approval of the final version of the manuscript; drafting and editing of the manuscript; collection, analysis, and interpretation of data; critical review of the literature; critical review of the manuscript.

Rafael Lanaro: Approval of the final version of the manuscript; collection, analysis, and interpretation of data; critical review of the manuscript.

Ana Maria Roselino: Approval of the final version of the manuscript; conception and planning of the study; collection, analysis, and interpretation of data; effective participation in research orientation; intellectual participation in the propaedeutic and/or therapeutic conduct of the studied cases; critical review of the manuscript.

## Conflicts of interest

None declared.
