# Supplementary material for: Measurement of pesticides in hair samples from pemphigus foliaceus and pemphigus vulgaris patients in Southeastern Brazil
Source: An Bras Dermatol. 2023 May 17;98(5):644–50. doi: 10.1016/j.abd.2022.10.010 (PMC10404494; doi:10.1016/j.abd.2022.10.010)
Supplement: Supplementary file 1 [file mmc1.docx]

**ABD-D-22-00416_ Supplementary Material**

**Supplementary Table 1** Analytical parameters of the target compounds.

|  | **Retention Time** | **Target ion (m/z)** | **Fragments (m/z)** | | | **Detection Limit** | **Determination Limit** |
| --- | --- | --- | --- | --- | --- | --- | --- |
| **Pesticides** | **RT (minutes)** | **M1** | **M2** | **M3** | **M4** | **LOD (pg/mg)** | **LOQ (pg/mg)** |
| Dichlorvos | 4.618 | 109 | 185 | 79 | 47 | 2.5 | 5 |
| Alpha-BHC | 6.893 | 181 | 183 | 219 | 217 | 2.5 | 5 |
| Terbufos | 6.914 | 57 | 231 | 97 | 103 | 5 | 5 |
| Diazinon | 6.951 | 179 | 137 | 152 | 199 | 2 | 2 |
| Disulfoton | 7.038 | 88 | 89 | 97 | 142 | 2 | 2 |
| Methyl parathion | 7.335 | 109 | 263 | 125 | 79 | 2.5 | 5 |
| Heptaclore | 7.408 | 100 | 272 | 274 | 270 | 2.5 | 5 |
| Fenitrotion | 7.504 | 277 | 125 | 109 | 260 | 5 | 5 |
| Malathion | 7.553 | 125 | 173 | 93 | 127 | 2 | 5 |
| Clorpirifos | 7.607 | 197 | 199 | 314 | 97 | 2 | 5 |
| Fention | 7.64 | 278 | 125 | 109 | 169 | 5 | 5 |
| Aldrin | 7.652 | 66 | 263 | 79 | 265 | 2.5 | 5 |
| Parathion | 7.66 | 97 | 109 | 291 | 139 | 2.5 | 5 |
| Methidation | 8.04 | 145 | 85 | 93 | 125 | 2.5 | 5 |
| Endosulfan | 8.159 | 195 | 197 | 241 | 237 | 2.5 | 5 |
| Ethinon | 8.542 | 231 | 153 | 97 | 125 | 2.5 | 5 |
| Endrin | 8.633 | 67 | 345 | 250 | 347 | 2.5 | 5 |
| DDT | 8.794 | 235 | 237 | 165 | 199 | 5 | 5 |
| Hexazinone | 8.823 | 171 | 83 | 128 | 71 | 5 | 5 |
| Keto endrin | 9.09 | 67 | 317 | 315 | 319 | 2.5 | 5 |

LOQ, Limit Of Quantification; LOD, Limit Of Detection; M/Z, Mass divided by charge of the target compound; M1, Main ion (quantifier), M2, 3 and 4, Minority ions (qualifiers).
